# Livestock movement informs the risk of disease spread in traditional production systems in East Africa

**DOI:** 10.1038/s41598-021-95706-z

**Published:** 2021-08-12

**Authors:** Divine Ekwem, Thomas A. Morrison, Richard Reeve, Jessica Enright, Joram Buza, Gabriel Shirima, James K. Mwajombe, Tiziana Lembo, J. Grant C. Hopcraft

**Affiliations:** 1 Boyd Orr Centre for Population and Ecosystem Health, Institute of Biodiversity, Animal Health & Comparative Medicine, College of Medical, Veterinary and Life Sciences, University of Glasgow, Glasgow, UK; 2 School of Computing Science, University of Glasgow, Glasgow, UK; 3 Nelson Mandela African Institution of Science and Technology, Arusha, Tanzania; 4 Department of Livestock, Serengeti District Council, Ministry of Livestock and Fisheries, Serengeti, United Republic of Tanzania

**Keywords:** Ecology, Diseases, Risk factors

## Abstract

In Africa, livestock are important to local and national economies, but their productivity is constrained by infectious diseases. Comprehensive information on livestock movements and contacts is required to devise appropriate disease control strategies; yet, understanding contact risk in systems where herds mix extensively, and where different pathogens can be transmitted at different spatial and temporal scales, remains a major challenge. We deployed Global Positioning System collars on cattle in 52 herds in a traditional agropastoral system in western Serengeti, Tanzania, to understand fine-scale movements and between-herd contacts, and to identify locations of greatest interaction between herds. We examined contact across spatiotemporal scales relevant to different disease transmission scenarios. Daily cattle movements increased with herd size and rainfall. Generally, contact between herds was greatest away from households, during periods with low rainfall and in locations close to dipping points. We demonstrate how movements and contacts affect the risk of disease spread. For example, transmission risk is relatively sensitive to the survival time of different pathogens in the environment, and less sensitive to transmission distance, at least over the range of the spatiotemporal definitions of contacts that we explored. We identify times and locations of greatest disease transmission potential and that could be targeted through tailored control strategies.

## Introduction

Livestock are central to the world economy and form the basis of livelihoods throughout rural Africa. The widespread incidence of economically devastating infectious diseases in this region, however, directly threatens livestock production, health and survival^1,2^. Recognition that livestock movement is an important driver of infectious disease transmission and spread has stimulated research efforts to understand how and where livestock move in different settings^3–5^. In Africa, livestock movements are largely motivated by a need for animals to access resources (e.g. grazing and watering) to ensure their survival. Livestock often travel several kilometres each day to reach communal resource areas where extensive mixing of herds and contacts between animals occur, with considerable implications for pathogen transmission and subsequent disease spread to other areas^6–9^. Contact patterns may also be influenced by livestock management practices, resource distribution^7,10,11^ and trade^12^. Measuring “contacts” is therefore crucial to elucidating disease dynamics and devising appropriate management approaches^13^. For a given pathogen with a particular mode of transmission, a key goal is to understand where and when, in a given landscape, hosts are most likely to come into contact and thus to propagate disease. Identifying disease transmission flashpoints is important because these locations could allow for bespoke and targeted interventions to be applied^14^, which can be especially important in resource-limited settings.

Measuring contact in many African settings is challenging due to the highly unregulated and undocumented nature of movements^8,10,15^. In addition, livestock populations do not mix homogeneously because herd movement is influenced by a range of factors including seasonality, herd size, availability of and proximity to resource areas, and household wealth^10,11,16^. Depending on the pathogen of interest, the definition of a contact relevant for disease transmission varies. For example, pathogens that are only infectious for a short period of time (e.g. a few hours or a day) in the environment, particularly in tropical settings (e.g. foot-and-mouth disease (FMD) and peste des petits ruminants (PPR) viruses) require close contact for optimal transmission between herds^17,18^. They will therefore spread across a population very differently from pathogens that have prolonged environmental survival (e.g. *Bacillus anthracis*)^19–21^. Other pathogens require close contact at a fine-scale (e.g. with infectious materials), but can also transmit at longer distances (e.g. *Coxiella burnetii*)^22^. The latter is especially relevant for herd-to-herd transmission. Therefore, both complexities in the mode of transmission, and temporal and spatial components need to be taken into account when quantifying contact^23^.

Regardless of the definition of contact, contacts will be more likely in some areas and under some conditions than others. For example, in the dry season when surface water is only available at a few sites, we would expect livestock to aggregate near water holes, thus elevating contact risk^9^. Moreover, some areas may be disproportionately risky relative to the amount of time that individual animals spend in or near them. One approach to understanding this relative risk is to account for the set of locations that animals use within their landscape and to compare these locations to those where contacts actually occurred. This conditional approach is conceptually similar to the widely used resource selection functions applied in wildlife ecology that identify animal habitat preferences^24^, and it provides a generalisable way to predict contact risk across landscapes with different spatial arrangements of resources.

Traditional methods to understand livestock movements and measure contacts, such as questionnaires or focus groups discussions and livestock market purchase permits^12,25,26^, are labour-intensive and limited by observational errors and recall biases^27^. In particular, these methods do not capture fine-scale livestock movements such as between-herd interactions, which are vital for understanding the spread of diseases and predicting outbreak sizes. Global Positioning System (GPS) loggers, in contrast, provide detailed information on livestock movements at fine temporal and spatial scale which allow for the estimation of the timing and location of contacts between animals^28,29^. Despite the usefulness of these devices, their application to research on livestock movement in sub-Saharan Africa is limited. Studies of this type in Kenya and Cameroon revealed that cattle moved several kilometres each day and that herds come into contact relatively frequently, likely resulting in increased disease spread^8,9^. These studies focused on pastoralist systems, yet fine-scale patterns of movement and contact in the most dominant livestock management system in sub-Saharan Africa, agropastoralism^30,31^, is urgently needed to understand disease spread and to inform management strategies tailored to these and similar production systems.

In this study, we use low-cost GPS data loggers to characterise livestock movements around communal areas of aggregation in a typical agropastoral community in East Africa. First, we describe parameters of movements by exploring trajectories of mobility and herding characteristics at shared resource areas. Movement patterns include daily movements, areas where livestock spend long periods of time, the speed of movement at various times of the day, and shared routes within and between villages. Second, we categorise between-herd absolute contact rates across a range of spatiotemporal scales. More specifically, given heterogeneities in contact relevant to disease transmission, we explore how different spatiotemporal scales influence observed rates of contact. Third, we describe the relative probability of contact to explore risks of disease spread for different resource areas. Specifically, in order to understand the role such areas may play in driving disease dynamics we investigate how the probability of a contact varies depending on the temporal and spatial window used to define it. Finally, we use our data to identify hotspot locations based on varying spatiotemporal definitions of contact and assess the extent to which hotspots change given the contact category. Our approach provides novel insights into how pathogens with different spatiotemporal modes of transmission pose risks for livestock disease spread in endemic areas.

## Methods

### Study area

**Figure 1:**
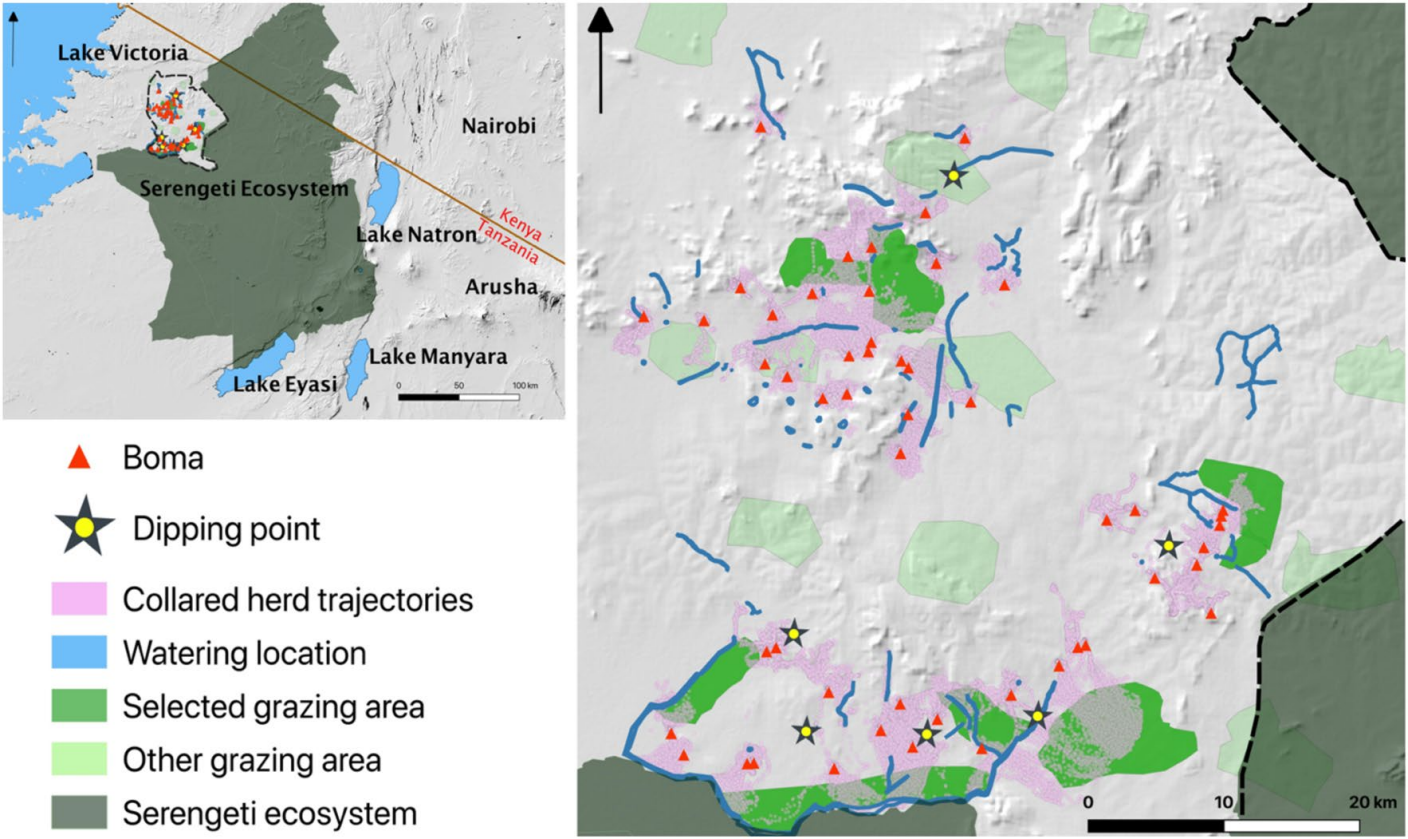
Map of the study area in northern Tanzania (left) showing cattle resource areas (grazing, watering and dipping points) that was determined from a previous study^11^, bomas and collared herds’ movement trajectories. The map was developed in quantum geographic information system (QGIS), version 3.16.8^32^.The region’s climate has alternating wet and dry seasons that affect the distribution and availability of water and forage resources for livestock^33^.

The study was conducted in the Serengeti District, an area of ~ 11,000 km^2^ located in northern Tanzania near the boundary of the Serengeti Ecosystem (Fig. 1). The district is inhabited by multi-ethnic permanent households who practise agropastoralism and keep livestock in enclosures at night. For the purpose of this paper, we define a permanent household with a livestock enclosure as a “boma”.

### Deployment of GPS collars

We deployed GPS loggers on 52 herds (one cow per herd) located throughout the study area (Fig. 1 and Supplementary Fig. S1) between November 2017 and June 2019. Loggers recorded locations every 15 min.

Briefly, the selection of herds for collaring was based on villages’ locations relative to livestock resource areas, and farmers’ stated use of those areas, determined through a community-level mapping exercise from a previous study^11^. In each village using these resource areas, we deployed collars on two herds from different households (*see* Supplementary Methods for details of herd selection).

### Data analyses

We used the GPS data to: (a) describe basic parameters of cattle movements, including distance, speed and diel pattern, (b) measure the absolute number of contacts among collared cattle, (c) examine the relative contacts between collared cattle to assess the potential for disease transmission, and identify hotspot locations based on varying spatiotemporal definitions of contact. All analyses were performed in the R environment^34^.

#### Parameters of cattle movements

Descriptive statistics were used to summarise metrics of herd movement. The step-length was defined as the trajectory between consecutive GPS fixes every 15 min. Speed was calculated for each step and averaged hourly. The daily distance travelled was the cumulative distance moved between fixes for each 24-h period. Maximum daily displacement was the distance between the farthest point and the animal’s boma. Generalised linear mixed models (GLMMs) were used to investigate the effects of cumulative rainfall in the previous month (i.e. monthly rainfall), herd size, and their interaction, on the total daily distance travelled and the maximum daily displacement. Monthly rainfall data for the study area were extracted from the ‘CHIRPS’ rainfall product which blends satellite data of cloud density with ground station rain gauges to generate 0.05 × 0.05 degree grid cells^35^. Monthly rainfall was averaged across the bounding box of the study area. Rainfall in the month preceding each cattle location was included as a predictor in the analysis. Total daily distances travelled were log-transformed to improve normality. We included a random effect corresponding to the unique herd identifier and inferred significance based on whether 95% confidence intervals overlapped zero.

#### *Measurement of absolute contacts between collared cattle*.

We explored contact rate across spatial and temporal scales to understand how this might change the probability of transmission for different pathogens. The spatial scales ranged from 50, 100, 200, 500 and up to 1000 m between each pair of herds, and the temporal scale ranged from 1 h, 1 day (24 h) and up to 1 week (168 h). We estimated contact rates for each combination of spatial and temporal scale (n = 15 total), which ranged from fine (i.e. 50 m and 1 h) to coarse (i.e. 1000 m and 1 week) scales. Field validation suggested that a herd of 50 grazing cattle generally occupied an area of about 100 m in diameter, while herds of 100 cattle occupied an area of approximately 200 m in diameter (Ekwem, *unpublish. data*). Given that the size of the herds tracked in our study ranged from 30 to 500 cattle, we considered spatial scales of 50 to 200 m as representing close contact (Supplementary Fig. S2).

At each scale, we measured pairwise contact rates (i.e. frequency of contacts per hour) for all possible combinations of collars. Given that collars were not all deployed throughout the same period of time, we only measured contact rates over the time period when both collars within a given pair were active.

To investigate how herd contact rates varied as a function of the spatial proximity between each herd’s boma, we measured ‘distance between bomas’ as the straight-line distance between each pair of sampled bomas, a value that ranged between 1 and 59 km (Fig. 1). Our modelling approach involved understanding the relative effect sizes of spatiotemporal definitions and distances between boma on pairwise contact rates. We fit GLMMs using the glmmTMB package^36^ and assumed contact rates were beta-distributed. We fit three models, one for each temporal scale (i.e. 1 h, 1 day and 1 week). In each model we included the spatial component (a categorical variable: 50 m, 100 m, 200 m, 500 m and 1000 m) and the pairwise distance between each herd’s boma (a continuous variable, measured in km) as explanatory variables, and treated the two IDs of each collar involved in a contact as a random intercept.

#### Expected contact rates

To understand the expected contact rate for cattle and allow comparisons to similar livestock production systems in East Africa, contact rates were standardised based on the number of other collared herds within a specified area. Standardised contact rates were calculated as the ratio of the sum of all pairwise contact rates of herds whose bomas were located within a given distance of one another to the total number of tracked herds in that area (including those that had no contacts). For example, herds from bomas located within 5 km, 10 km and 15 km cover an area of 19.6, 78.5 and 176.7 km^2^, respectively. If there were 25 contacts between herds whose bomas were within 5 km of each other and there were 8 herds tracked, the standardised expected contact rate would be 3.13 contacts per herd over the time period (i.e. 25/8). The probability of contacts between herds from bomas at distances > 15 km was extremely low (Fig. S5). This allowed us to estimate expected contact rate per cattle herd for every spatiotemporal definition of contact, given known population size of cattle in western Serengeti.

#### Relative contact probability between collared cattle

In order to determine if contacts were associated with specific features in the landscape, for example water sources or dipping points, we modelled the probability of contact across the movement trajectories of collared animals. For each observed pairwise contact (i.e. a “case” point), we randomly selected five additional “control” points from each of the individual trajectories involved in the contact. We considered control points to be locations where contacts had not occurred and these were selected without replacement from periods when both GPS loggers were active. This “case–control” design allowed for the analysis of relative contact probability using a generalized linear mixed effect model with a binomial error (case contact = 1 and control no-contact = 0). The approach is conceptually analogous to resource selection functions, which are a class of models used to quantify habitat suitability in animal movement studies^24^. Predictor variables included monthly rainfall and herd size, as well as distance to grazing areas, water holes, livestock dips, salt licks and bomas. Values of herd size and distance to home boma were selected at random from one individual within the pair for each contact. The IDs of each pair of individuals involved in a contact were included as random intercepts.

The analysis was performed for nine spatiotemporal combinations. The selected spatial and temporal scales represent a range, from fine- (i.e. contact for spatial component of 50 m and temporal component of 1 h) to coarse-scale (i.e. spatial component of 500 m and temporal component of 1 week) definitions of contact. Each model was built separately and model selection was performed with Akaike Information Criterion (AIC)^37^. Model comparison was performed using *summs* function in the *jtools* package in R^38^. Model predictions of relative contact probability were used to identify hotspots of disease transmission risk (i.e. locations where predicted contact probabilities are high). To illustrate this approach, we developed maps of relative probability for a single collared herd from western Serengeti, based on the top candidate model for each spatiotemporal definition of contact. This allowed us to examine the extent to which the identified hotspots changed given the spatiotemporal definitions of contact.

### Ethics statement

Overall permission to conduct this research, including the use of cattle for GPS-telemetry studies, was granted by the Tanzanian Commission for Science and Technology (permit numbers 2016-93-NA-2016-87 and 2017-284-NA-2016-87). Permission for research in communities was obtained from relevant local and district authorities, including veterinary offices and village leadership. In addition, individual cattle owners were informed about the background and objectives of the research through a Participant Information Sheet developed for this purpose. Participation was voluntary and written and/or verbal informed consent was obtained before proceeding. Handling of cattle for collaring purposes was minimal and was undertaken by fully trained staff, including veterinarians and livestock officers, according to local veterinary rules and informed by the UK Animals (Scientific Procedures) Act 1986 (amended 2012). Cattle were restrained carefully, calmly and humanely to ensure the safety of handlers and animals. In all cases, owners were involved in restraining their cattle to ensure minimal distress to the animal. Collared cattle were monitored regularly to ensure that the collars caused no injury or discomfort.

## Results

Data from 50 cattle herds were used in the analyses and consisted of 901,883 GPS fixes generated from November 2017 to May 2019. Because of occasional malfunctions in devices (e.g. battery or technical failure of the GPS device), not all devices were active for the entire study period. Two collars were lost entirely and therefore data could not be retrieved. All livestock owners confirmed that the tracked cattle remained in the herd and that animals were herded together throughout the study period, suggesting that collaring one cow was generally sufficient to track the entire herd.

### Parameters of cattle movements

Cattle moved near and within communal resource areas, including those allocated to grazing, watering and dipping (Fig. 1). There was a considerable degree of spatial overlap between herds at or near these resource areas.

#### Speed, step length, timing and distance of cattle movements

**Figure 2:**
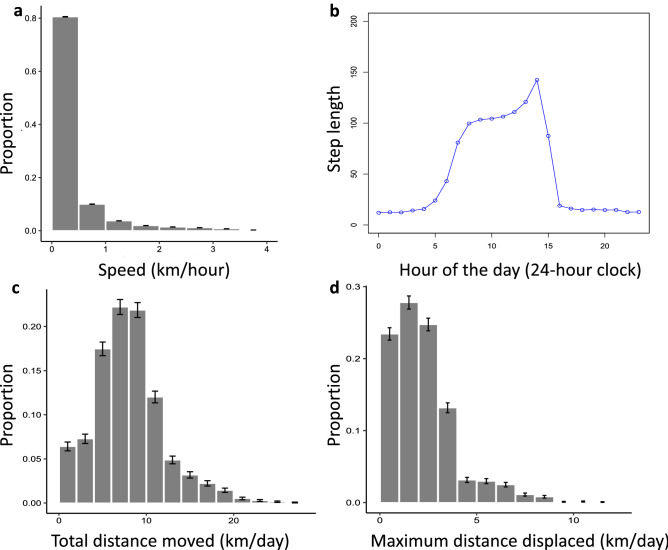
Speed, timing and distance of movements observed among collared cattle. (**a**) The speed cattle moved during the study period; (**b**) the median step length across the day (i.e. median distance moved every 15 min within the hour); (**c**) the total daily distance GPS collared cattle moved (i.e. the sum of the distance of all 15-min relocation intervals); and (**d**) the maximum daily distance cattle were displaced from their home bomas. Error bars denote standard error of the distribution.

Although cattle moved as fast as 4.00 km/hour, the median speed was 0.09 km/hour (IQR: 0.04– 0.37 km/hour) (Fig. 2a). There was wide variation in the observed step lengths of movement at the 15-min fix interval across the diurnal cycle (Fig. 2b). Fifteen-minute median step lengths for all cattle ranged from < 10 m, when most individuals were resting at their bomas, to 147 m, when animals were actively travelling. Individuals were most active in daylight, between 5:00 to 20:00 h, with peak movements around 13:00 -14:00 h every day (Fig. 2b). Cattle were displaced from their bomas by a median distance of 1.94 km (IQR: 1.05 – 2.97 km) (Fig. 2c and Supplementary Fig. S3) but travelled for a median total daily distance of 7.72 km (IQR: 5.39-9.99 km; Fig. 2d and Supplementary Fig. S3).

#### Factors influencing distance travelled

**Figure 3:**
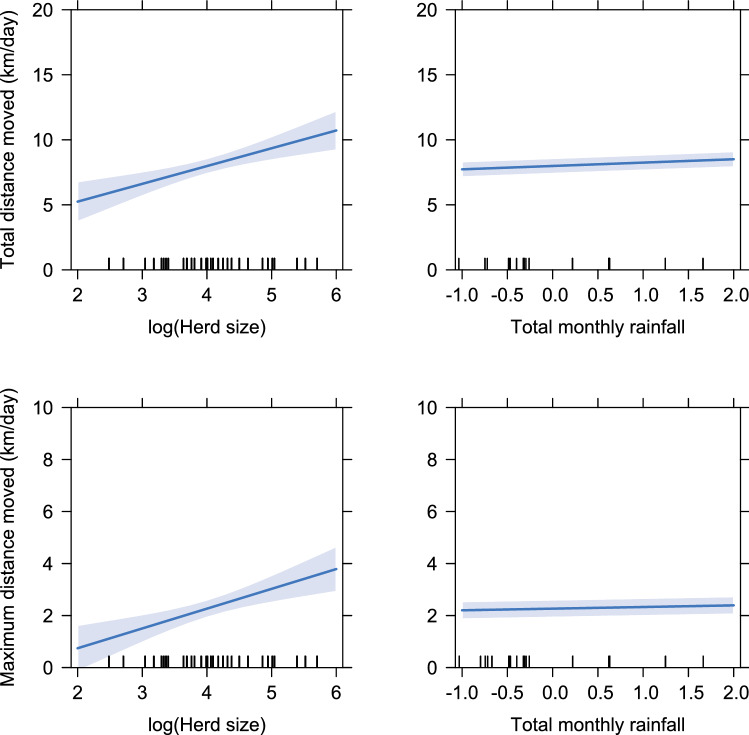
Herd size (log-transformed) and cumulative rainfall in the previous month (scaled and centered) increase the daily movement distance and displacement of cattle. Lines represented predicted effects from the GLMMs. Shaded areas are the 95% confidence intervals.

The daily distance travelled and the maximum distance displaced from the boma increased with log herd size, and marginally increased with monthly rainfall (Fig. 3). Specifically, the total daily distance increased by an estimated 1.46 km (CI: 0.92–2.01 km) for each log-unit increase in herd size and by 0.26 km (CI: 0.19–0.32 km) for each unit increase in scaled monthly rainfall, with no interaction between herd size and rainfall (*β* = 0.02, CI: −0.05–0.07) (Fig. 3). Similarly, the maximum daily displacement increased by 0.79 km (CI: 0.36–1.22) for each log-unit increase in herd size and by 0.10 km for each scaled unit increase in rainfall, with a significant positive interaction between herd size and rainfall (*β* = 0.10, CI: 0.05–0.14).

### Absolute pairwise contact rates among collared cattle

Generally, contacts occurred at all times of the day but with the highest frequency around 10:00 −15:00 h, when cattle were most active, for all spatiotemporal definitions. The only exception was for the smallest spatial scale (50 m) when there were considerable high contacts in the early morning (4:00 am) (Supplementary Fig. S4).

#### Factors affecting absolute pairwise contact rates

**Figure 4:**
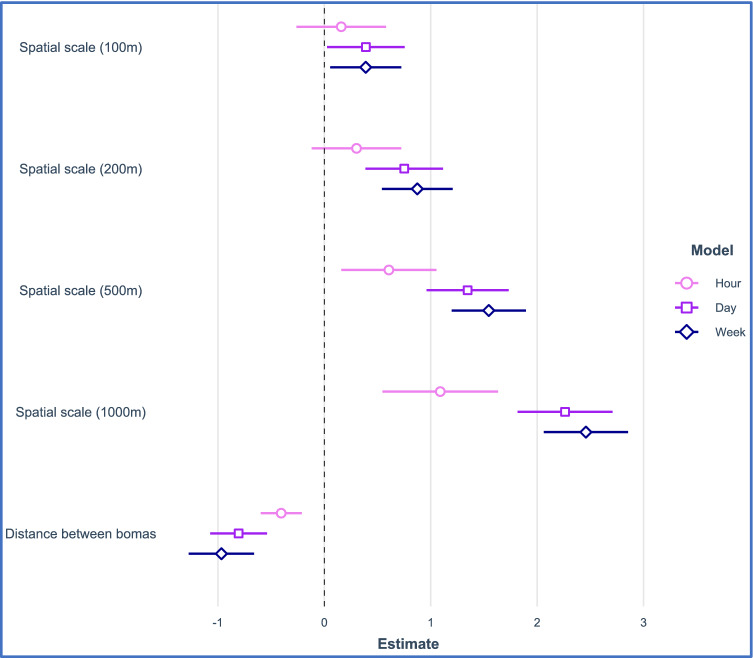
The relative effect size of temporal and spatial definitions of contact, and distances between bomas, on the pairwise contact rate (hr^−1^). Symbols denote estimated coefficients (± 95% CI) from three separate models. The effect size of spatial scale coefficients are relative to the reference level of 50 m in all models.

Overall, the time window of contact (temporal scale) had a larger effect on absolute contact rates than the spatial scale across the range of spatiotemporal values investigated (Fig. 4). Coarse spatiotemporal definitions of contact were generally associated with increased pairwise contact rates of collared cattle (Fig. 4). However, contact rates did not differ significantly at small spatiotemporal scales (e.g. within 1-h at 50–200 m proximity) (Fig. 4). Contacts decreased as the distance between cattle’s bomas increased (Fig. 4), up to a maximum distance of ~ 17 km, beyond which no contacts were observed (Supplementary Fig. S5).

#### Expected contact rates

The expected pairwise cattle contact rates depended on the spatiotemporal scale used in the definition of contacts (Supplementary Fig. S6). For example, after standardising for cattle herds from bomas located within 10 km of one another (i.e. an area of 78.5 km^2^), the expected contact rate was 0.015 h^−1^ when contacts were defined as occurring within 200 m within an hour. Yet, the expected contact rate was nearly four times higher (0.059 h^−1^) when contacts were defined as occurring within 200 m within a day (Supplementary Fig. S6). Details of the expected contact rates for all spatiotemporal definitions are included in Supplementary Fig. S6.

### Relative probability of contact

**Figure 5:**
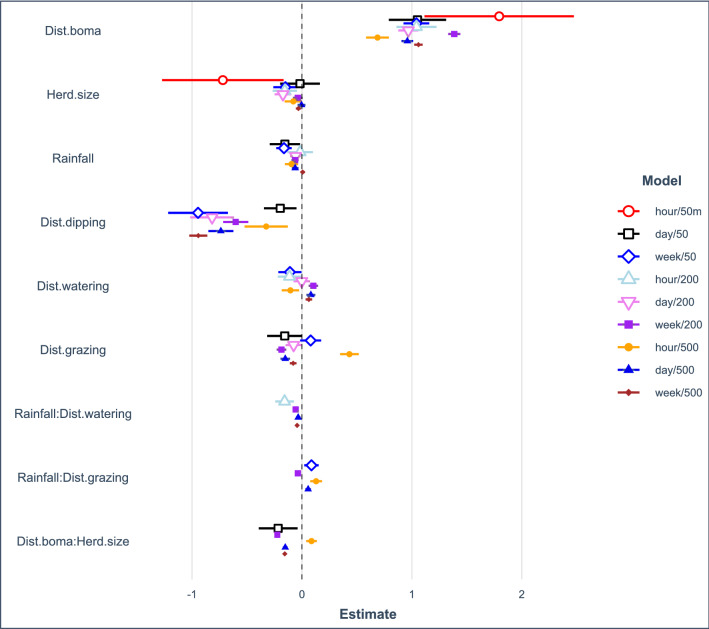
Factors affecting the relative probability of contact in cattle change in their effect sizes across a range of spatiotemporal scales. Variables tested in each model are indicated on the Y-axis, with symbols denoting the mean effect size (lines denote 95% C.I.) of variables retained in the top candidate model at each spatiotemporal scale of contact.

We investigated landscape variables that changed the contact probability among collared cattle across nine spatiotemporal scales, using a separate model for each scale (Fig. 5). The direction and magnitude of effects were not consistent for all variables across models, suggesting that drivers of contact depended on the spatiotemporal scale of contacts. Details of each model including variables that remained in the final model and summary outputs are included in Supplementary Tables S1-3 (*c.f.* Fig. 5).

The variable with largest effect across all models was distance to bomas, with higher probability of contact as cattle moved farther from their bomas. Large herds had lower contact probability than small herds, and the interaction term between herd size and distance to boma suggested relatively fewer contacts in large herds as cattle move farther from bomas. Generally, rainfall was an important predictor of relative contact probability, with fewer contacts during wetter periods, though the effect of rainfall decreased with larger spatiotemporal scales of contact (Fig. 5).

Distance to dipping was the only resource variable with a consistent effect across all models, where higher contact probability occurred in proximity to livestock dips (Fig. 5). For distances to watering and grazing, the direction of effects depended on the spatiotemporal scale of contacts. At small spatiotemporal scales, the contact probability was higher closer to watering points, while the opposite was the case for large spatiotemporal scales. However, the interaction between rainfall and distance to watering points was negative, suggesting that as rainfall increased distance to watering became a less important driver of contact probability. In the majority of models, relative contact probability was higher closer to grazing areas. For one spatiotemporal scale of contact (within 500 m in an hour) relative contact probability increased as cattle moved away from grazing areas. Generally, the interaction between rainfall and distance to grazing areas was positive, suggesting that away from grazing areas contacts were more likely when rainfall was high (Fig. 5).

**Figure 6:**
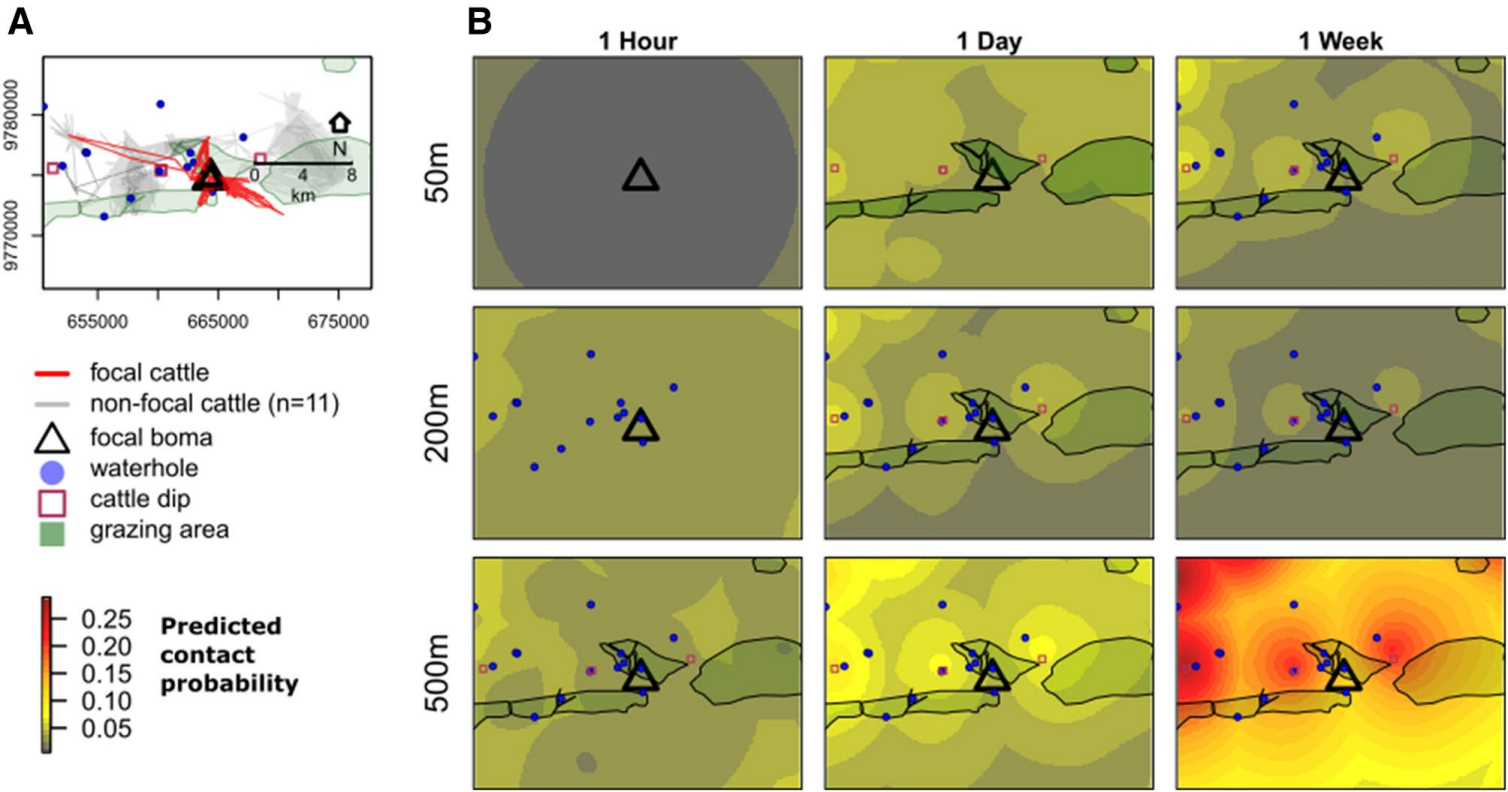
Predicted contact risk for a focal cattle across an agropastoral community in northern Tanzania. (**a**) GPS trajectories of focal cattle and neighboring cattle (n = 11) in relation to communal resource areas (grazing, watering and dipping points). (**b**) Relative contact risk within the same landscape is based on model predictions for a single cow originating from its home boma (black triangle) for three spatial (50 m, 200 m and 500 m) and three temporal (1 h, 1 day, 1 week) scales of contact (*c.f.* Fig. 5). Extent of the mapped area is limited to the prediction range of the focal cattle, within 10 km buffer to display risk around nearby resource areas. The high risk near waterholes and dipping areas is based on the predicted values from the population-level model, so may not have been visited by the focal cattle during the study period. Resource areas are mapped if they were retained in the top candidate model. Monthly rainfall was set to median value for all predictions. Log-herd size was set to the value of the focal cattle herd (103 individuals, untransformed), representing a relatively large herd for this dataset.

Model coefficients were used to generate predictions of relative contact risk across the study area. In Fig. 6, we use a single focal animal to illustrate how these predictions provide spatially-explicit information on relative contact probability, given the distribution of resources and the spatiotemporal definition of contact. For instance, contact risk increases away from bomas and near to cattle dips across most spatiotemporal scales of contact, yet resource areas also generate complex spatial patterns in relative risk because of clustering in the distribution of resources (Fig. 6b). Further, these predictive maps show how the relative probability of contact increases at large spatiotemporal scales of contact, and how areas have different relative contact risks at each scale (Fig. 6b).

## Discussion

Understanding the spatial patterns and drivers of animal movement is a crucial first step to controlling disease spread^4^. Our study provides novel information about where, how and when cattle move in a region beset by endemic pathogens^2,39,40^. Because contacts occur heterogeneously through time and space, interventions targeting areas and times of high contact risk could effectively break the chain of transmission across wide areas. We found that cattle herds had the highest probability of contact at dipping sites, far from their bomas, in small herds and during periods of low rainfall, indicating that transmission of all pathogens may be particularly elevated under these conditions (Figs. 5, 6). Nonetheless, cattle spent most of their time in other areas (i.e. near bomas or in grazing areas) where the direction and magnitude of effect of spatiotemporal scale on contact rates varies. This suggests that interventions for different pathogens in these systems will likely require a consideration of scale of transmission and be tailored to particular pathogens. Overall, our study provides a framework for risk-based livestock disease control approaches for the most dominant management systems in sub-Saharan Africa.

Daily movement patterns of cattle in pastoral and agropastoral settings in sub-Saharan Africa largely reflect the distribution of shared resources, which determines the distance animals move each day and the probability of contacting each other. Our results are similar to those reported in other regions of Africa, suggesting broadly comparable patterns of daily displacement. For instance, cattle in our agropastoral study area travel to grazing, watering and dipping locations that are ~ 4 km from their bomas and primarily during daylight hours (Fig. 2). Similarly, in Kenya, cattle in the pastoral Mara and Ol Pajeta regions move less than 6 km from their bomas and movements peak around 12:00–14:00 h each day^9,41^. Despite the predominance of short-distance daily movements, we observed occasional long-distance movements (i.e. up to 12 km), particularly by larger herds. Transhumant cattle in Cameroon also moved up to 23 km/day for short periods, while relocating to seasonal grazing areas on the edge of the Sahel, though in most observations (86%) they moved less than 5 km/day^8^. Although we observed no contacts among cattle from bomas > 17 km apart (Supplementary Fig. S5), regardless of how contact was defined, infrequent long-distance movements by large herds may provide a conduit for disease transmission between villages^42^. Indeed, larger herds actually had a lower relative probability of contact across spatiotemporal scales (Fig. 5), which may reflect the fact that large herds were more likely to move to areas away from other collared cattle, either because they were moving outside the study area, or because they had exclusive use of particular areas, whereas smaller herds that were mostly moved around bomas mixed more frequently. While interventions (e.g. vaccination or quarantine) targeting small herds would address local disease events, particularly within villages, halting larger-scale transmission requires an understanding of livestock pathways enabling inter-village connectivity and strategies tailored to herds driving these processes.

A key difference between the movement of cattle in agropastoral and pastoral systems lies in the seasonal variation of daily movement. In our study, agropastoralists move their herds farther in the wet compared to the dry season, while the opposite has been reported for pastoralists^8,9,41^. During the wet season, agropastoralists cultivate crops near their homesteads, which increases competition for space and displaces cattle to reserved grazing areas far from cultivated land^11^. During the dry season, particularly in the early period, cattle graze harvested fields around the homestead and tend to move short distances each day. In our study, although individual herds travelled more (marginally) in the wet compared to the dry season, there were more contacts following low rainfall periods when resources were typically scarce (Fig. 5). Similarly, a previous study has shown that more villages were connected at shared resource areas during dry spells, which resulted in higher contacts^11^. This suggests a higher disease risk in the dry compared to wet seasons in agropastoral management systems.

Translating movements into contact between individuals is challenging because the definition of a “contact” depends on the distance at which pathogens can travel in space, and the time period that pathogens survive, or mature to an infectious state, in the environment. Most studies that attempt to measure contact, however, focus only on a single scale. Here, we show that pairwise contact rates between cattle herds generally increase with broader spatiotemporal definitions of contact. Yet, there was no difference at spatial scales between 50 m, 100 m and 200 m for a temporal scale of one hour, suggesting these scales are functionally equivalent definitions of contact. Thus, we define “close contact” as proximity of livestock herds within 200 m in any given hour, which would be applicable to multiple disease systems and vital for understanding infectious disease spread in traditionally managed herds. However, given that herds tracked in our study ranged in size from 30 to 500 cattle, for households with herds of < 30 cattle, a meaningful spatial scale of close contact would likely be less than 200 m. Furthermore, the relative effect of temporal scale on the contact rate was not the same at all spatial scales (Fig. 4). For instance, there was a large difference in the number of contacts between 1 h, 1 day and 1 week at all spatial scales greater than 200 m. Thus, the temporal window that defines a contact has a greater influence on contact frequency than the spatial component, at least across the range of values in our study. Again, this is an important consideration when designing interventions to break transmission for specific pathogens.

Understanding how contact varies as a function of spatial and temporal scales enables us to hypothesise how transmission risk may vary as a function of a pathogen’s mode of transmission and its stability in the environment. For example, a coarse-scale definition of contact is relevant for understanding the transmission risk of pathogens that are stable in the environment. In contrast, a fine-scale definition applies to pathogens that, for optimal transmission, require hosts to be relatively close to each other in both space and time. Pathogens with multiple transmission modes, which is the most likely situation, might have risks associated with both coarse and fine-scale contacts.

Estimating the expected total number of contacts between livestock in an area illustrates the potential for disease transmission through a region. For example, consider an area with a 10 km radius (i.e. 314 km^2^)—in our study area this would contain about 4 villages, each having about 250 livestock-keeping households. For short-lived pathogens requiring close contact for transmission, a meaningful contact could be defined as being within 200 m within the same hour. Using the parameters from our results, the contact rate between collared cattle was observed to be 0.015 h^−1^ (Supplementary Fig. S6), suggesting that across all animals in the area, given the density of cattle herds, we would expect at least 1 contact every 4.76 h. However, for pathogens that spread by airborne transmission, a meaningful contact could be defined as being within 500 m within the same hour. In this case, using our observed contact rate of 0.059 h^−1^ between collared cattle (Supplementary Fig. S6), we estimate a contact occurring every 40 min across all cattle in the area. This illustration provides a unique perspective about the relative speed at which different pathogens could spread through an area. The next question is, given the movement patterns of livestock, where do these contacts occur and are there specific areas in the landscape that could be targeted?

Identifying where in a given landscape inter-herd contacts are most likely to occur is challenging in observational studies as inferences will be strongly affected by sampling design (i.e. which individuals have GPS collars and how many collars are deployed). We overcome this issue by estimating a relative contact probability that accounts for both the locations where contacts occur and where they *could* have occurred. This conditional approach shows that locations with high contact probability tend to expand as the spatiotemporal scale of contact increases, but this pattern is not consistent across all resources (Fig. 5). For instance, contact probability was high when herds were close to shared resources such as pasture and water, but the effect of distance to water on the probability of contact switched from negative to positive as the temporal and spatial scale increased from fine to coarse resolution (Fig. 5). This suggests that distance to water may pose different levels of risk depending on the pathogen’s mode (e.g. direct versus indirect) transmission.

Identifying areas of high congregation and potential contact offers opportunities for preventative interventions, especially in settings where large-scale disease mitigation efforts, for instance mass livestock vaccination, are limited, such as our study context. Sharing of communal resources is considered a major driver of infectious disease transmission in traditionally managed livestock husbandry systems^42–44^. In our study, areas of increased contact included dips and water points. Livestock tend to congregate infrequently at dips, twice a month in most cases^11^. However, these gatherings are exceptionally large and intense because the dipping schedule is restricted to a few events per month. This results in a significantly higher probability of contacts across all spatiotemporal scales and suggests that these areas pose very large relative transmission risks for most types of pathogens. An initial approach to reduce high livestock congregations may be to keep dips open for more days per month and to introduce a strict rota system by household. The risk of transmission around waterholes is offset by precipitation; under high rainfall conditions when access to water is unrestricted, there are few contacts between herds and transmission risk is reduced. Repeated contacts for short periods of time also occurred at watering points (i.e. for minutes to an hour, once per day generally between 13:00 and 15:00 h), which would favour pathogens with short survival times and close-contact transmission. Fewer encounters around watering points for long contact duration suggest lower risks for pathogens (such as *B. anthracis*) with prolonged environmental survival, especially when precipitation levels are high. In contrast, herders access multiple grazing areas each day and tend to avoid other herds to minimise mixing and theft^11^. Therefore, sharing pasture results in more herds but fewer close-encounter contacts. Sharing grazing areas at distinct nonoverlapping times would thus favour the transmission of pathogens with long survival in the environment or that can be transmitted from afar. Thus, for volatile pathogens, transmission risks are likely higher around shared dips or watering points. Reducing opportunities for contacts at these locations would lower exposure risks and the number of herd infections Although contact patterns can be used to infer transmission and infection risks^9,23^, disease spread is influenced by complex interactions between biological and ecological factors relevant to each pathogen, only some of which are outlined here. For example, for diseases that require an arthropod vector (e,g, African trypanosomiasis transmitted by tsetse flies) or an intermediate host (e.g. echinococcosis or other parasitic diseases requiring complex transmission cycles involving intermediate and definitive hosts), pathogen transmission can occur independently of direct contact amongst individuals of the species of primary concern. Similarly, water- or food-borne infections do not require close contact for transmission. Environmental and climatic drivers, such as exceptionally high and low rainfall or soil characteristics, critically influence the ecology of pathogens with an environmental phase, e.g. *B. anthracis*. Fomites also contribute to within and between herd transmission of FMD and occasionally, wind-borne particles of FMD virus can facilitate the spread of the disease over long distances. Consequently, our findings should be interpreted with caution in the knowledge that contacts were defined broadly, and further sources of heterogeneity are likely. In addition, our study focused on inter-herd contacts, but intra-herd dynamics may play an important role in the speed of disease spread. Furthermore, the mobility of infected animals may be impacted in ways that change contact risks, but this heterogeneity was not captured in our dataset. Ultimately, there is a need for further studies that integrates infection and transmission surveillance with herd mobility information to establish the extent to which contact risk predicts infection of different pathogens.

A fruitful area for future research would be to compare contact rates across a broad spectrum of production systems, for example from pastoral to peri-urban smallholder systems. The transition of many communities towards urbanization could change the patterns of contact, particularly because peri-urban livestock owners have alternate sources of income and often more wealth than rural subsistence farmers. Furthermore, many sub-Saharan governments are encouraging the expansion of mechanised agriculture and agro-industry (high production dairies, supplemental feeding with hay and silage, and large abattoirs to supply the export market). These cultivated sites will overlap with traditional livestock keeping areas and could dramatically change livestock movements and disease dynamics, which will require tailored mitigation strategies. Another important area for future work would be to test how the spatial pattern and availability of critical resources could reduce mixing and disease spread while improving herd management. For instance, a simulation experiment in which the number of contacts is estimated based on the fine scale movements of multiple herds in silico would allow researchers to explore the effects of adding, removing or changing the availability of resources such as dips, water holes or additional pasture at different locations in the landscape.

Finally, linking data on fine-scale movements of livestock with epidemiological data generated in near real time during outbreak investigations would help validate our models. It would also provide valuable information on locations and times that might drive transmission risks and that could therefore be targeted through tailored interventions. Specifically, for infectious diseases that transmit over limited distances and require close/direct contact with infectious animals or materials (e.g. FMD), combining livestock movement data and epidemiological modelling of outbreak information would enable us to identify central transmission nodes (e.g. shared dips and watering locations) and times of highest congregation (e.g. periods of low rainfall), and to evaluate mitigation scenarios through movement restrictions or vaccination at these key points. In areas where large-scale vaccination programmes are impractical, this information could shape the development of locally-acceptable interventions (e.g. use of acaricide hand spray and watering livestock using water troughs) that could reduce transmission at these locations and times, hence help to flatten the epidemic curve. This type of approach would also be valuable for informing and prioritising resources for vaccination. For example, previous work on FMD in these settings ^2^ suggests that dominant viral serotypes move slowly, one at a time, in waves across the landscape, and that this pattern may be consistent across East Africa. Spatial models of disease spread informed by timely detection of outbreaks at the serotypic level would enable us to target vaccination ahead of the wave of infection using monovalent vaccines which are more widely available than polyvalent formulations.

## Conclusion

The widespread movements and herd contacts we report here reflect potentially high disease transmission risks among traditionally managed livestock in East Africa because of their reliance on shared resource areas. The standardising and scaling up of contact rates have allowed us to estimate expected contact rates between herds in field settings, which could be generalised to other similar areas across the region. The classification of between-herd contacts across a range of temporal and spatial scales has improved our understanding of the specific risk associated with different pathogen modes of transmission. This information, combined with locations where herds interact the most, provide valuable insights for devising targeted control strategies for different pathogens. Our findings are broadly generalisable and demonstrate the need for disease-specific interventions for livestock raised in traditionally managed systems.

## Supplementary Information

Supplementary Information.
