# Supplementary material for: Livestock movement informs the risk of disease spread in traditional production systems in East Africa
Source: Sci Rep. 2021 Aug 12;11:16375. doi: 10.1038/s41598-021-95706-z (PMC8361167; doi:10.1038/s41598-021-95706-z)
Supplement: Supplementary file 1 — Supplementary Information. [file 41598_2021_95706_MOESM1_ESM.pdf]

Livestock movement informs the risk of disease spread in traditional production systems in East Africa

Divine Ekwem[1], Thomas A. Morrison[1*], Richard Reeve[1], Jessica Enright[2], Joram Buza[3], Gabriel Shirima [3], James K. Mwajombe[4], Tiziana Lembo[1**], J. Grant C. Hopcraft[1**]

*Corresponding author
**These authors contributed equally

[1]Boyd Orr Centre for Population and Ecosystem Health, Institute of Biodiversity, Animal Health & Comparative Medicine, College of Medical, Veterinary and Life Sciences, University of Glasgow, Glasgow, United Kingdom

[2]School of Computing Science, University of Glasgow, Glasgow, United Kingdom

[3]Nelson Mandela African Institution of Science and Technology, Arusha, Tanzania

[4] Department of Livestock, Serengeti District Council, Ministry of Livestock and Fisheries, United Republic of Tanzania

Supplementary Materials

Supplementary Methods.

Herd selection for Global Positioning System collaring study of cattle

We selected herds using a four-stage selection process:

1. Selection of resource areas: Six replicate grazing areas were randomly selected from a list of the largest 18 shared grazing areas in Serengeti District. All selected grazing areas were <1 km from at least one major watering point (Fig. 1, main text).

2. Selection of villages: For each selected grazing area, all villages that grazed in the area (n = 26) were included in the study.

3. Selection of herds: In each village, one large and one small herd were selected randomly for Global Positioning System (GPS) collar deployment from the list of all bomas, on condition that each boma was >1 km apart. The number of cattle in each herd was classified by size based on community perceptions as follows: large herds (≥90 cattle) and small herds (<90). Within villages, pairwise distances between bomas ranged from 1-5 km.

4. Selection of cattle for collaring: One healthy cow per herd was considered sufficient to track collective group movements [1].

GPS data retrieval and processing

Each sampled boma with collared cattle was visited every four weeks to retrieve data from the GPS devices. Owners were asked weekly if collared animals had remained with the herd to ensure the GPS data were representative of the herd's movement. Occasionally, collared cattle were sold, in which case another collar was deployed on a comparable individual within the same herd.

Collar data were pre-processed by excluding unrealistically high movements speeds (>20 km/hour) [2,3]. These errors occurred infrequently and likely related to GPS satellite coverage, or possibly transport of collars within a moving vehicle.

Supplementary Figures

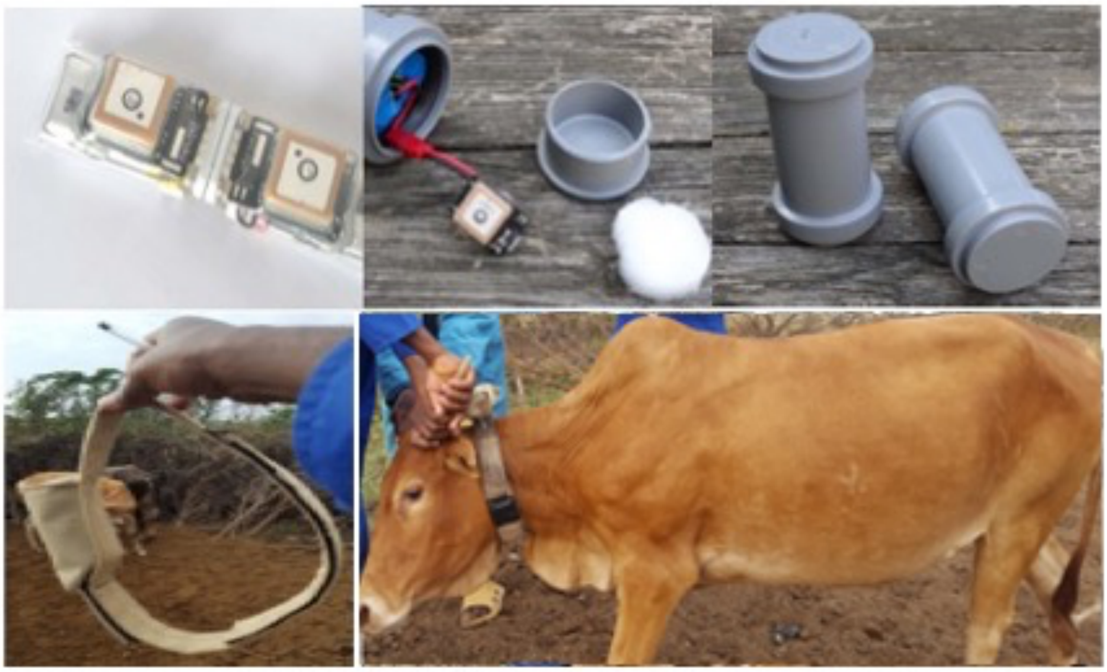

Figure S1. Pictures showing the components of Global Positioning System collars used in the study to track movements of individual cattle in a herd. Complete units were mounted on cattle using locally-produced collars.

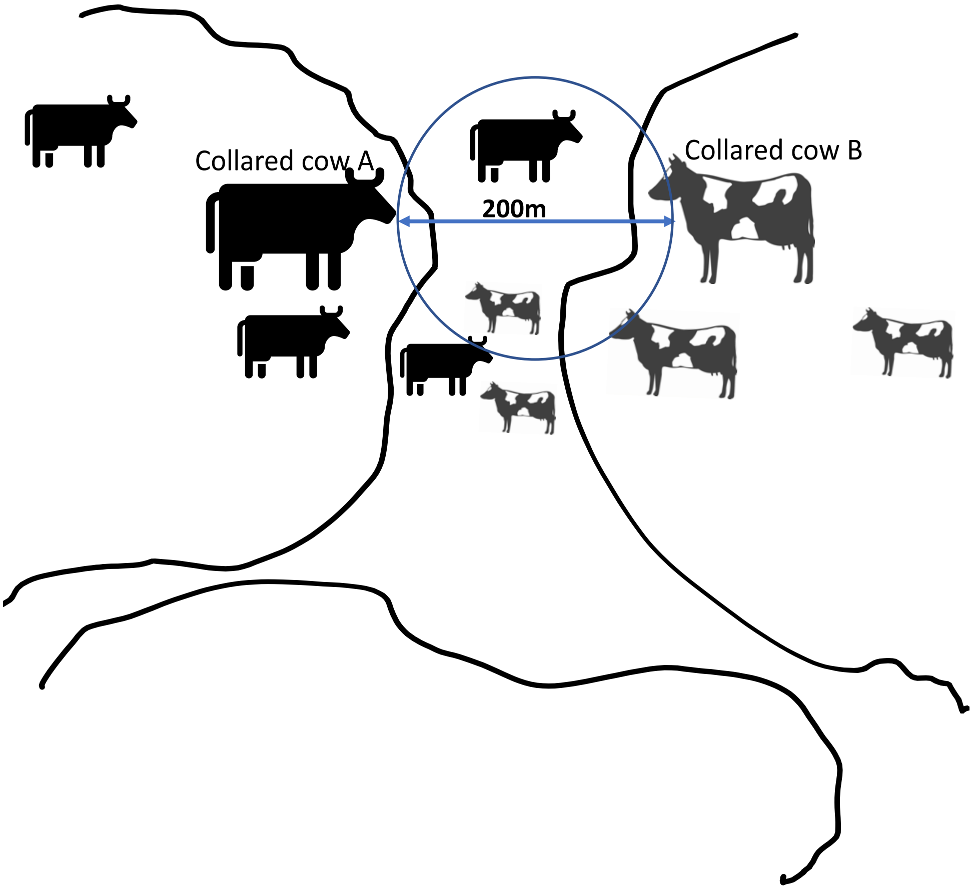

Figure S2. A schematic used to define contact of two herds A and B (black and black and white cattle, respectively). The blue circle is the spatial scale, which in this case has a buffer size of 200 meters in diameter (double arrow). The buffer size indicates the proximity of a contact, which in this case was defined as two GPS fixes from two different herds with location less than 200 metres apart within one hour. The figure also shows that, although the distance between the two collared cattle in their herds was 200 meters, other cattle from both herds were likely to be in the path (less than 200 m) of collared cattle A and B.

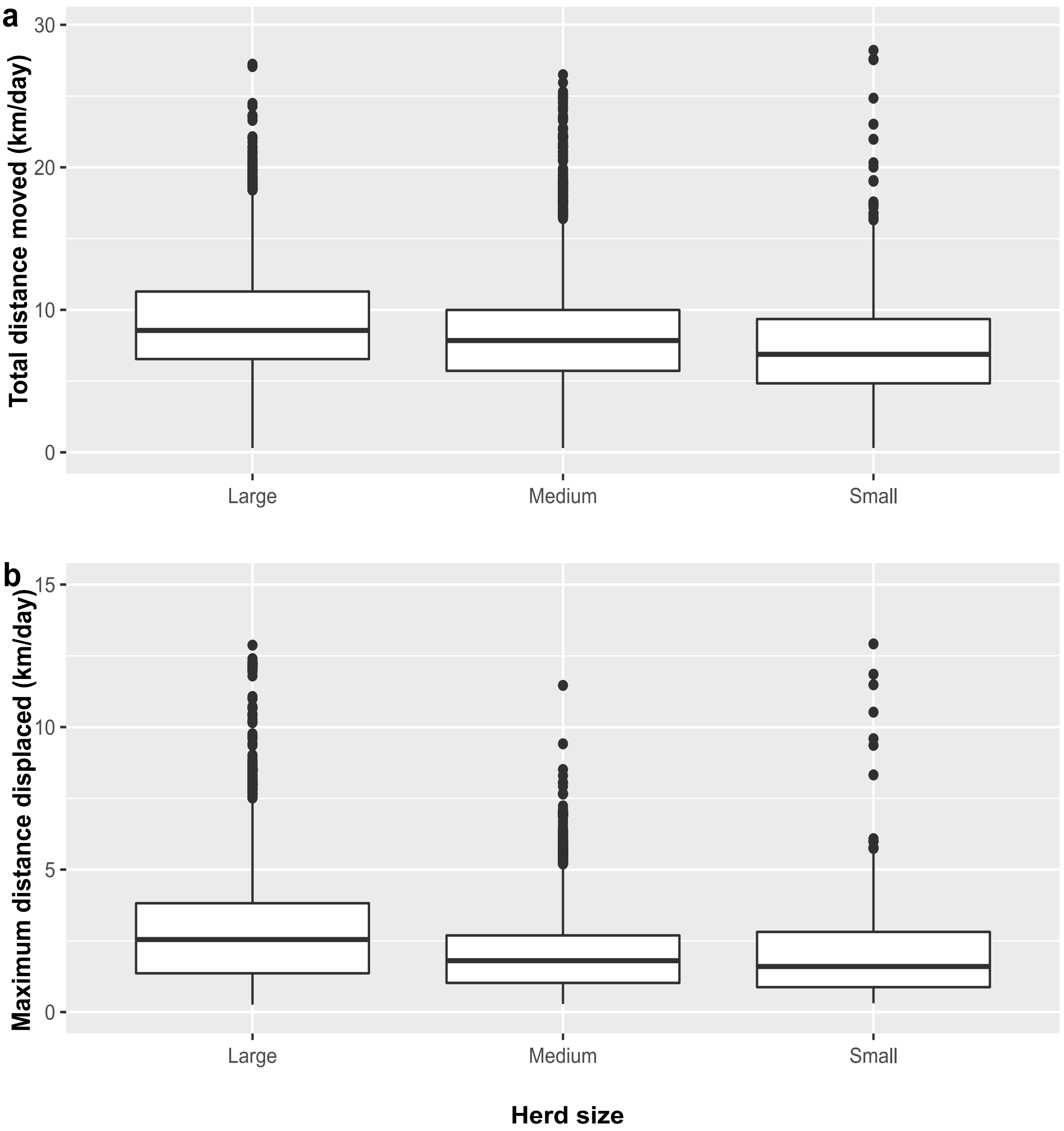

Figure S3. Distribution of the overall livestock herd mobility across herd sizes during the study period: (a) total distance moved daily and (b) maximum distance displaced from the home boma. Boxplots represent median and interquartile range (IQR). Herds were classified as: large (>90), medium (30-90) and small (<30).

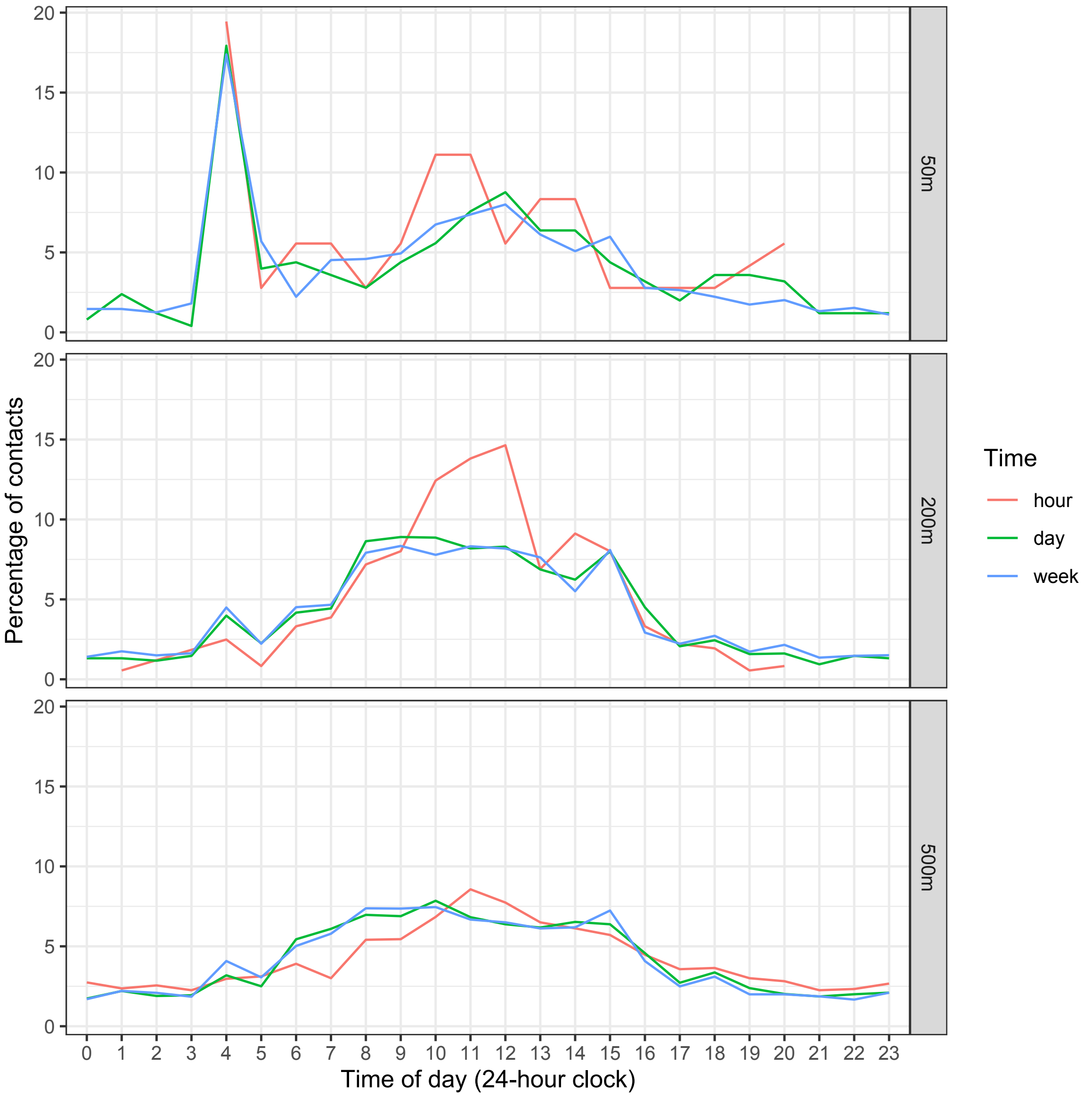

Figure S4. The proportion of contacts that occurred for each hour in the day. The spatial window (contact distance) was set at 50m, 200m and 500m. The temporal window (duration of contact) ranged from one hour to one week.

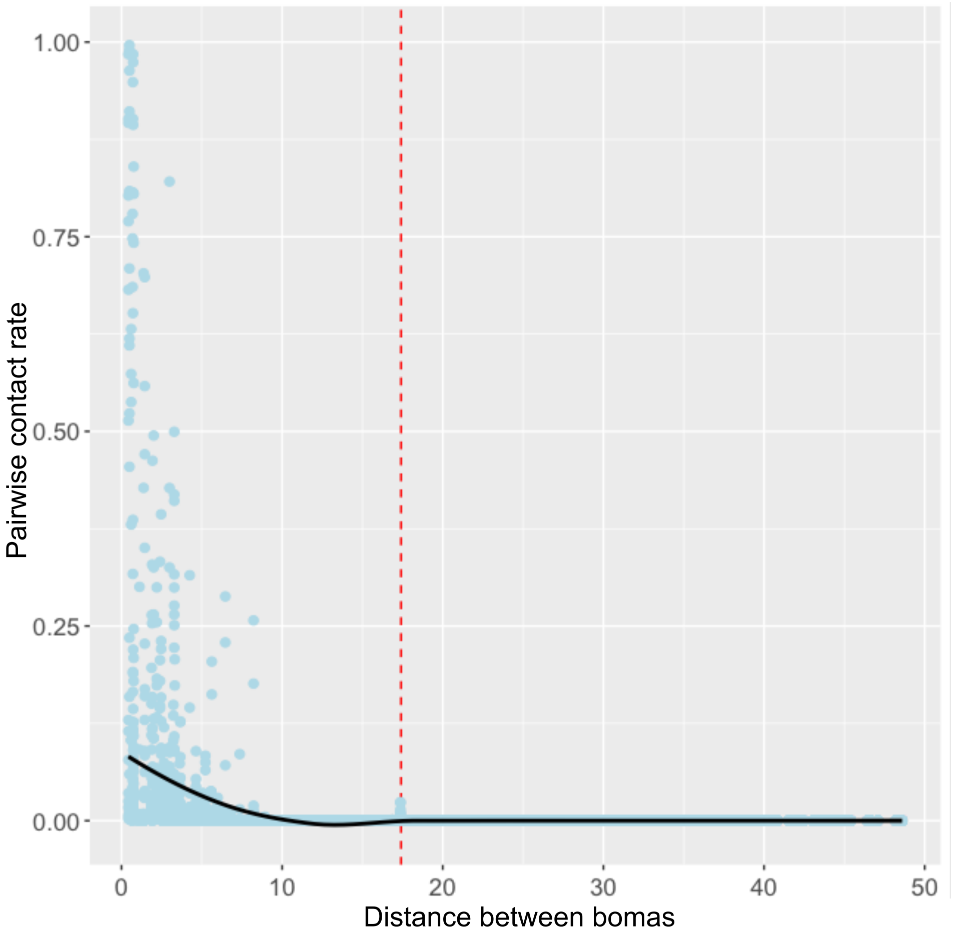

Figure S5. Pairwise contact rates across all spatiotemporal definitions of contacts decline with distance (km) between pairs of cattle's bomas. The contact rate between a pair of collared cattle was measured as the proportion of time they were in contact given a contact definition. The black fitted line is the regression line. The dashed reference line shows the distance beyond which collared cattle had no contact based on all contact defintions.

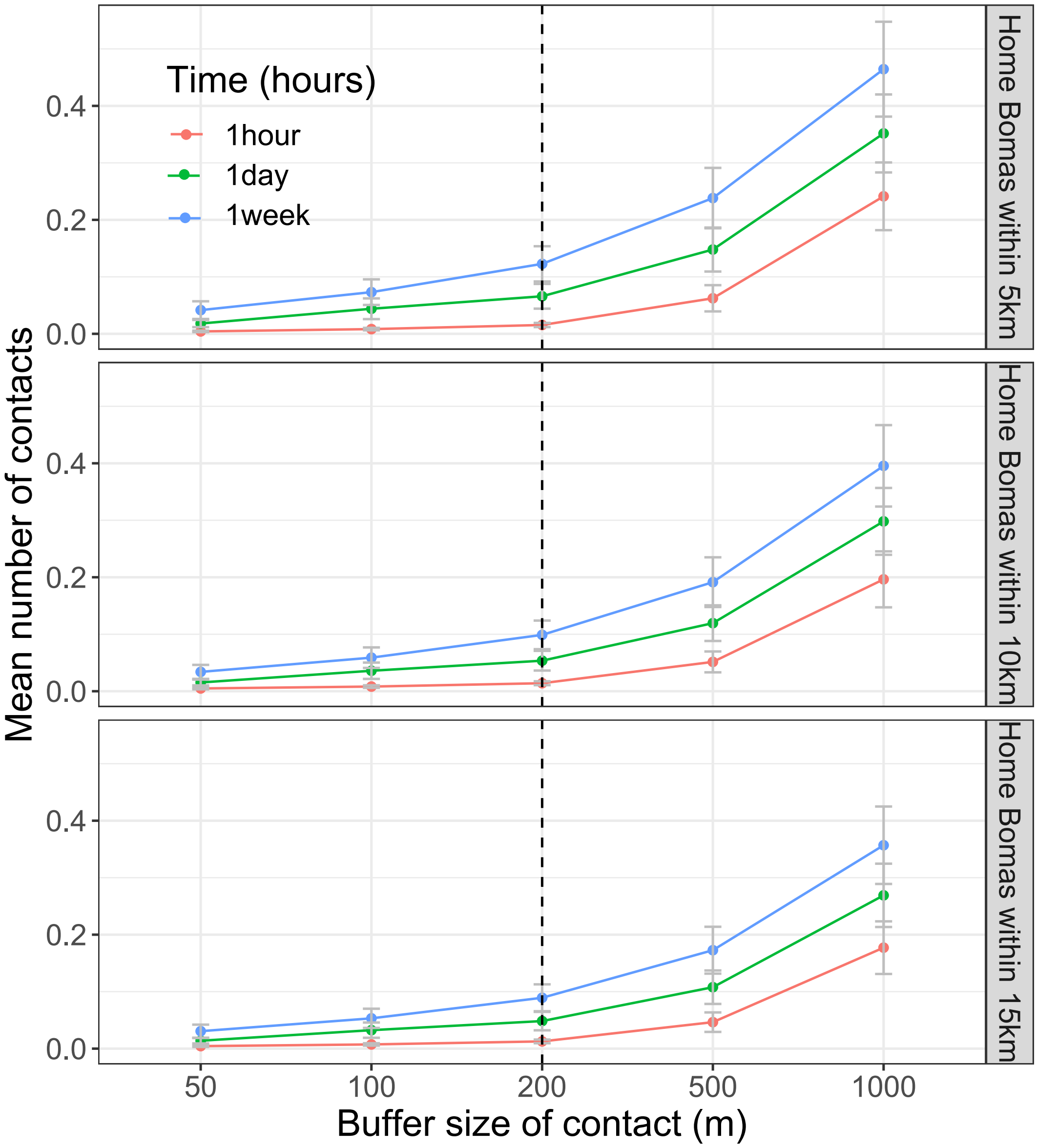

Figure S6. Mean standardised contact rates of cattle increase with spatiotemporal windows (i.e. buffer size and lagged time). Error bars are the standard error of the mean. The spatial window (contact distance or buffer size) was set at 50, 100, 200, 500 and 1000 m. The temporal window (lagged time interval) ranged from 1 to 168 hours (i.e. one hour to one week). The reference line at 200 meters indicates the assumed close contact distance. Herds from home bomas located within 5, 10 and 15 km comprise an area of 19.6, 78.5, 176.7 km^2, respectively.

Supplementary Tables

Table S1. Details of the binomial regression models (i.e. contact spatial window of 50 metres and temporal windows of 1 hour to 1 week), which examined factors that affect the probability of herd contacts relative to resource areas that was measured across a range of spatiotemporal proximities. Models were tested inividually and continuous variables were scaled to mean zero and one standard deviation (SD). CI denotes confidence intervals.

Model	Fixed effects of final model	Model parameters	
		Estimate	Odds Ratio (95% CI)
Spatiotemporal proximity of 50 metres in one hour	Distance to boma	1.80	6.02 (3.05 - 11.88)
	Herd size	-0.72	0.49 (0.28 – 0.85)
Spatiotemporal proximity of 50 metres in one day	Distance to boma	1.05	2.86 (2.20 - 3.71)
	Herd size	-0.02	0.98 (0.82 - 1.18)
	Rainfall	-0.16	0.86 (0.75 - 0.98)
	Distance to dipping	-0.20	0.81 (0.68 - 0.96)
	Distance to grazing	-0.16	0.85 (0.73 - 1.00)
	Distance to boma: Herd size	-0.22	0.82 (0.71 - 0.95)
Spatiotemporal proximity of 50 meters in one week	Distance to boma	1.04	2.83 (2.52 - 3.18)
	Herd size	-0.15	0.86 (0.77 - 0.96)
	Rainfall	-0.16	0.85 (0.79 - 0.91)
	Distance to dipping	-0.95	0.39 (0.30 - 0.51)
	Distance to watering	-0.11	0.90 (0.81 – 1.00)
	Distance to grazing	0.08	1.08 (0.98 - 1.19)
	Rainfall: Distance grazing	0.09	1.09 (1.02 - 1.16)

Table S2. Details of the binomial regression modes (i.e. contact spatial window of 200 metres and temporal windows of 1 hour to 1 week), which examined factors that affect the probability of herd contacts relative to resource areas that was measured across a range of spatiotemporal proximities. Models were tested inividually and continuous variables were scaled to mean zero and one standard deviation (SD). CI denotes confidence intervals.

Model	Fixed effects of final model	Model parameters	
		Estimate	Odds Ratio (95% CI)
Spatiotemporal proximity of 200 metres in one hour	Distance to boma	1.04	2.83 (2.36 - 3.40)
	Herd size	-0.16	0.86 (0.77 – 0.96)
	Rainfall	-0.02	0.98 (0.87 – 1.11)
	Distance to watering	-0.11	0.89 (0.80 – 0.99)
	Rainfall: Distance watering	-0.16	0.85 (0.78 – 0.93)
Spatiotemporal proximity of 200 metres in one day	Distance to boma	0.97	2.63 (2.40 - 2.88)
	Herd size	-0.17	0.84 (0.78 - 0.91)
	Rainfall	-0.06	0.94 (0.90 - 0.99)
	Distance to dipping	-0.82	0.44 (0.36 - 0.54)
	Distance to watering	-0.01	1.00 (0.93 - 1.07)
	Distance to grazing	-0.08	0.93 (0.86 - 1.00)
Spatiotemporal proximity of 200 meters in one week	Distance to boma	1.39	4.00 (3.79 - 4.22)
	Herd size	-0.04	0.96 (0.92 - 1.01)
	Rainfall	-0.06	0.94 (0.91 - 0.97)
	Distance to dipping	-0.60	0.55 (0.49 - 0.61)
	Distance to watering	0.10	1.11 (1.06 – 1.16)
	Distance to grazing	-0.19	0.83 (0.79 - 0.87)
	Rainfall: Distance watering	-0.60	0.94 (0.92 - 0.96)
	Rainfall: Distance grazing	-0.04	0.96 (0.94 - 0.99)
	Distance to boma: Herd size	-0.22	0.80 (0.78 – 0.82)

Table S3. Details of the binomial regression models (i.e. contact spatial window of 500 metres and temporal windows of 1 hour to 1 week), which examined factors that affect the probability of herd contacts relative to resource areas that was measured across a range of spatiotemporal proximities. Models were tested inividually and continuous variables were scaled to mean zero and one standard deviation (SD). CI denotes confidence intervals.

Model	Fixed effects of final model	Model parameters	
		Estimate	Odds Ratio (95% CI)
Spatiotemporal proximity of 500 metres in one hour	Distance to boma	0.69	1.99 (1.79 – 2.21)
	Herd size	-0.08	0.92 (0.85 – 1.00)
	Rainfall	-0.10	0.91 (0.86 – 0.97)
	Distance to dipping	-0.33	0.72 (0.59 – 0.88)
	Distance to watering	-0.11	0.90 (0.83 – 0.97)
	Distance to grazing	0.43	1.54 (1.42 – 1.68)
	Rainfall: Distance to grazing	0.13	1.14 (1.08 – 1.20)
	Distance to boma: Herd size	0.09	1.09 (1.04 – 1.14)
Spatiotemporal proximity of 500 metres in one day	Distance to boma	0.96	2.61 (2.47 – 2.75)
	Herd size	-0.00	1.00 (0.96 – 1.03)
	Rainfall	-0.06	0.94 (0.91 – 0.97)
	Distance to dipping	-0.77	0.47 (0.41 – 0.52)
	Distance to watering	0.08	1.08 (1.04 – 1.13)
	Distance to grazing	-0.15	0.86 (0.82 – 0.90)
	Rainfall: Distance to watering	-0.03	0.97 (0.95 – 0.99)
	Rainfall: Distance to grazing	0.06	1.06 (1.03 – 1.09)
	Distance to boma: Herd size	-0.15	0.86 (0.84 – 0.88)
Spatiotemporal proximity of 500 meters in one week	Distance to boma	1.06	2.89 (2.78 – 3.00)
	Herd size	-0.03	0.97 (0.95 - 0.99)
	Rainfall	0.01	1.01 (0.99 - 1.03)
	Distance to dipping	-0.39	0.39 (0.36 - 0.42)
	Distance to watering	0.06	1.07 (1.03 – 1.10)
	Distance to grazing	-0.08	0.92 (0.90 - 0.95)
	Rainfall: Distance to watering	-0.04	0.96 (0.94 - 0.97)
	Distance to boma: Herd size	-0.15	0.86 (0.84 - 0.87)

References

1. Moritz, M., Galehouse, Z., Hao, Q. & Garabed, R. B. Can One Animal Represent an Entire Herd? Modeling Pastoral Mobility Using GPS/GIS Technology. *J. Hum. Ecol.* **40**, 623–630 (2012).

2. Gamo, R., Rumble, M., Lindzey, F. & Stefanich, M. GPS Radio Collar 3D Performance as Influenced by Forest Structure and Topography. *Biotelemetry* **15**, 464–473 (2001).

3. Gerlach, F. L. & Jasumback, A. E. Global Positioning System Canopy Effects Study. *US Department of Agriculture Forest Service, Technology and Development Center: Missoula, MT, USA.* (1989).
